# Supplementary material for: Correlation of pitching velocity with anthropometric measurements for adult male baseball pitchers in tryout settings
Source: PLoS One. 2022 Mar 17;17(3):e0265525. doi: 10.1371/journal.pone.0265525 (PMC8929570; doi:10.1371/journal.pone.0265525)
Supplement: Attachment — Submitted filename: Response to Reviewers.docx [file pone.0265525.s002.docx]

PONE-D-21-08727

Correlation of pitching velocity to the anthropometric measurements for male adult baseball players

PLOS ONE

Dear Dr. Chiu,

Thank you for submitting your manuscript to PLOS ONE. After careful consideration, we feel that it has merit but does not fully meet PLOS ONE’s publication criteria as it currently stands. Therefore, we invite you to submit a revised version of the manuscript that addresses the points raised during the review process.

Please emphasize the novelty of the study, which both reviewers pointed. Please improve the clarify in methods/results. Thorough literature review as well as discussion are suggested as well.

Please submit your revised manuscript by Jul 11 2021 11:59PM. If you will need more time than this to complete your revisions, please reply to this message or contact the journal office at plosone@plos.org. When you're ready to submit your revision, log on to https://www.editorialmanager.com/pone/ and select the 'Submissions Needing Revision' folder to locate your manuscript file.

Please include the following items when submitting your revised manuscript:

A rebuttal letter that responds to each point raised by the academic editor and reviewer(s). You should upload this letter as a separate file labeled 'Response to Reviewers'.

A marked-up copy of your manuscript that highlights changes made to the original version. You should upload this as a separate file labeled 'Revised Manuscript with Track Changes'.

An unmarked version of your revised paper without tracked changes. You should upload this as a separate file labeled 'Manuscript'.

If you would like to make changes to your financial disclosure, please include your updated statement in your cover letter. Guidelines for resubmitting your figure files are available below the reviewer comments at the end of this letter.

If applicable, we recommend that you deposit your laboratory protocols in protocols.io to enhance the reproducibility of your results. Protocols.io assigns your protocol its own identifier (DOI) so that it can be cited independently in the future. For instructions see: http://journals.plos.org/plosone/s/submission-guidelines#loc-laboratory-protocols. Additionally, PLOS ONE offers an option for publishing peer-reviewed Lab Protocol articles, which describe protocols hosted on protocols.io. Read more information on sharing protocols at https://plos.org/protocols?utm_medium=editorial-email&utm_source=authorletters&utm_campaign=protocols.

We look forward to receiving your revised manuscript.

Kind regards,

Kei Masani

Academic Editor

PLOS ONE

Journal Requirements:

When submitting your revision, we need you to address these additional requirements.

1. Please ensure that your manuscript meets PLOS ONE's style requirements, including those for file naming. The PLOS ONE style templates can be found at

https://journals.plos.org/plosone/s/file?id=wjVg/PLOSOne_formatting_sample_main_body.pdf and

https://journals.plos.org/plosone/s/file?id=ba62/PLOSOne_formatting_sample_title_authors_affiliations.pdf

2. In your Data Availability statement, you have not specified where the minimal data set underlying the results described in your manuscript can be found. Regarding Data-sharing policy, it is unclear why 'No'.

PLOS defines a study's minimal data set as the underlying data used to reach the conclusions drawn in the manuscript and any additional data required to replicate the reported study findings in their entirety. All PLOS journals require that the minimal data set be made fully available. For more information about our data policy, please see http://journals.plos.org/plosone/s/data-availability.

The minimum dataset is uploaded and can be downloaded by all at

<https://drive.google.com/file/d/1XOI_smCGZYf75pq1HzLrkgM9MlUI_7W5/view?usp=sharing>

Upon re-submitting your revised manuscript, please upload your study’s minimal underlying data set as either Supporting Information files or to a stable, public repository and include the relevant URLs, DOIs, or accession numbers within your revised cover letter. For a list of acceptable repositories, please see http://journals.plos.org/plosone/s/data-availability#loc-recommended-repositories. Any potentially identifying patient information must be fully anonymized.

Important: If there are ethical or legal restrictions to sharing your data publicly, please explain these restrictions in detail. Please see our guidelines for more information on what we consider unacceptable restrictions to publicly sharing data: http://journals.plos.org/plosone/s/data-availability#loc-unacceptable-data-access-restrictions. Note that it is not acceptable for the authors to be the sole named individuals responsible for ensuring data access.

We will update your Data Availability statement to reflect the information you provide in your cover letter.

4. PLOS requires an ORCID iD for the corresponding author in Editorial Manager on papers submitted after December 6th, 2016. Please ensure that you have an ORCID iD and that it is validated in Editorial Manager. To do this, go to ‘Update my Information’ (in the upper left-hand corner of the main menu), and click on the Fetch/Validate link next to the ORCID field. This will take you to the ORCID site and allow you to create a new iD or authenticate a pre-existing iD in Editorial Manager. Please see the following video for instructions on linking an ORCID iD to your Editorial Manager account: https://www.youtube.com/watch?v=_xcclfuvtxQ

[Note: HTML markup is below. Please do not edit.]

Reviewers' comments:

Reviewer's Responses to Questions

Comments to the Author

1. Is the manuscript technically sound, and do the data support the conclusions?

The manuscript must describe a technically sound piece of scientific research with data that supports the conclusions. Experiments must have been conducted rigorously, with appropriate controls, replication, and sample sizes. The conclusions must be drawn appropriately based on the data presented.

Reviewer #1: Partly

Reviewer #2: No

2. Has the statistical analysis been performed appropriately and rigorously?

Reviewer #1: Yes

Reviewer #2: Yes

3. Have the authors made all data underlying the findings in their manuscript fully available?

The PLOS Data policy requires authors to make all data underlying the findings described in their manuscript fully available without restriction, with rare exception (please refer to the Data Availability Statement in the manuscript PDF file). The data should be provided as part of the manuscript or its supporting information, or deposited to a public repository. For example, in addition to summary statistics, the data points behind means, medians and variance measures should be available. If there are restrictions on publicly sharing data—e.g. participant privacy or use of data from a third party—those must be specified.

Reviewer #1: No

Reviewer #2: No

4. Is the manuscript presented in an intelligible fashion and written in standard English?

PLOS ONE does not copyedit accepted manuscripts, so the language in submitted articles must be clear, correct, and unambiguous. Any typographical or grammatical errors should be corrected at revision, so please note any specific errors here.

Reviewer #1: No

Reviewer #2: No

5. Review Comments to the Author

Please use the space provided to explain your answers to the questions above. You may also include additional comments for the author, including concerns about dual publication, research ethics, or publication ethics. (Please upload your review as an attachment if it exceeds 20,000 characters)

Reviewer #1: The authors prevent a valuable contribution to the fields of scouting, performance and coaching. That being said, I do feel these results have been produced elsewhere (like at Driveline baseball). There could be much more thorough review of literature, and tempered recommendations at the end of the paper. I have some suggestions for the authors to further investigate, primarily, including their 9 removed athletes that didn't meet the professional standard. I would like to see the author's data as well.

Line Abstract - Pitching, not Pithing

Corrected, thank you. We’ve also asked for help from professional English editing company and this manuscript is revised for proper style.

Line 3-5: I understand the author’s intention - to find the relationship between mechanics and velocity, but this is so insufficient when it comes to understanding the kinematics associated with velocity. In fact, only one paper is referenced regarding mechanics, when it is widely studied how workload, fatigue, mechanics, and anthropometry/skill contribute to velocity. Recommend citing a review paper such as Seroyer, S. T., Nho, S. J., Bach, B. R., Bush-Joseph, C. A., Nicholson, G. P., & Romeo, A. A. (2010). The kinetic chain in overhand pitching: its potential role for performance enhancement and injury prevention. Sports health, 2(2), 135-146.

Citation added, thank you.

Line 11 - “the extent to pitching velocity has not been fully explored” - please re-construct sentence. This does not make sense.

The sentence is re-constructed, thanks for reminding.

Line 13 - identify what changes in mechanics would cause change in velocity

We apologize for the overstatemen. The cited research is inter-individual, not intra-individual. Thank you for pointing it out.

The sentence is revised to “Studies have reported that better temporal parameters and kinematic parameters often infer to faster pitching velocity”

Line 16 - Furthermore, Driveline Baseball did a replication study on vertical jumps and the correlation to velocity. You do cite some other non-academic work, and I would recommend this being cited here.

https://www.drivelinebaseball.com/2016/09/examining-the-relationship-between-vertical-jump-force-and-velocity/

We do follow Driveline’s research and appreciate all the R&D done there. Citation added, thank you so much.

Line 22 - you are referencing Mercier here, could you please highlight the strength of the relationship in question? What was the r2 value?

Mercier gives no R2 value in the paper. That review is mainly about which research might be biased, and which parameters might truly be significant.

We’ve added another reference, Relationship Between Performance Variables and Baseball Ability in Youth Baseball Players Nakata, Hiroki (2013), which has more detailed correlation R value for physical tests.

Line 28 - what is your definition of “inexpensive field tests”?

We were hoping that the total cost is under USD $500.

My jump2 APP for measure CMJ is USD $18, and shows good correlation.

Line 38 - would like to know which previous studies are cited for each test

Citations added after each test accordingly, thank you.

Line 54 - Nine of the participants that were excluded from this study - did you have complete data for these athletes? Irrespective of their threshold for a professional standard, this could be a good data set to show the sensitivity of your model. If these athletes had very poor tests and the model predicted a very low velocity, this would only strengthen your model. Recommend these data be included.

The raw data, including the 9 excluded participants, is uploaded and can be downloaded at

<https://drive.google.com/file/d/1YO4NC2gGXitzGmlJaU3AhW2fMB6YSVvw/view?usp=sharing>

We re-run the model with 9 excluded participants and answer it together in the Line 160 question.

Line 66 - was it the same experimenter recording ROM for each participant? If not, what was the inter-rater reliability?

To finish all the tests in 2 hours, there were 3 experimenter recording ROM. Though we haven’t tested the inter-rater reliability, but all of experimenter are trained by Taiwan Athletic Trainers’ Society, by the same instructor and passed the certification exam.

Line 75 - see above comment. What is your definition of an inexpensive field test? How much was the Gym Aware hardware?

GymAware is USD $1,995. We were thinking that, comparing to Kistler force plate which costs over $40,000, under 2k is not expensive. It is still not cheap, we agree.

We agree that “inexpensive” is probably not the best word to describe it, “affordable” is used instead, plus option for under $1000 for all is added in the paper.

Line 93 - reference location of Rapsodo company

Reference updated, thank you.

Line 99 - R step{stats} - is this an error, or is this how the Stats package is referenced?

In R programming, the name of the package is enclosed by {}. To better deliver the message, the text has been modified accordingly. “A linear stepwise multiple regression analysis was performed using R with step function in {stats} package to investigate……….”

Line 106 - may relationships in biological systems are non-linear. Did you explore any non-linear terms in your regression modelling? Why not?

The ways to fit a nonlinear model are infinite. Without knowing a strong foundation of the starting values for the nonlinear algorithm from the literature, we preferred taking a conservative approach and fitting a linear model, which gives an ability to better interpret the R square and p value of each dependent variable.

Line 109 - many other studies have included BMI - would recommend this also be included in your model. While not perfect, it would give some context to large, muscular athletes.

Thank you for the suggestion. We ran an additional statistical analysis with BMI included in the regression model. The overall model fitting was not improved (R square = 0.223 versus 0.230 in the original model), thus we prefer keeping the model as it is (few predictors and higher R square value).

Fig1. The statistical result of the model presented in this study. (without BMI)

Fig2. The statistical result of the model with BMI included

Line 104 and Line 112 - Define VIF before using acronym

Thanks. We have edited the text accordingly. “Variance inflation factor (VIF) value was used to examine the multicollinearity”

Line 132 - Total COST of the equipment at 4500, not COSE

Corrected, thank you.

Line 132 - 4500 USD is definitely less expensive than a full motion capture lab, but it isn’t inexpensive in my opinion - particularly if you’re looking at youth athletes.

GymAware USD $1,995, can be replace by My Jump2 APP, which is USD $18 and has been validated by some research. https://147.197.131.104/handle/2299/21027.

A DIY laser gate measuring sprinting time costs around USD $120 can be used to measure sprinting time instead of Witty timing system. <https://create.arduino.cc/projecthub/Pablerdo/wireless-laser-gate-timing-system-for-track-and-field-ba8cd9>

or

Dashr timing system costs USD $350 <https://www.dashrsystems.com/products>

Line 148 - what is the “first move when pitching”?

Sentence is revised as follows, “In contrast to batting, the force does not have to be developed in the initial wind up move in pitching.”.

Line 160 - it sounds like you could erase the limitation of “not professional” by included the other 9 athletes who were not at the professional standard? Would like to see these data.

When drawing the tryout pitchers’ max velocity on a chart, there is a gap shown.

It looks like a good idea to make a cut at either 128km/h or 130km/h, since the data under 130km/h is very sparse.

Anyways, we still re-run the model with 9 removed pitchers added to the dataset, and here is the result.

Adjusted R2 is 0.161. And with the 9 low velo pitchers removed

Adjusted R2 is 0.230, with all the coefficients being significant.

Line 162 - I think this section of the paper is very important for the layman, particularly in an open source journal. However, I think the examples you have given here are requiring more context. For example, your model has an RSS of 3.57. Your two examples have an error of 2 and 3 km/h. This is very close to the model error, and I don’t know if it’s a strong enough of an example, in the lack of other data on mechanics for you to make your claims. A generic example of a “mechanical tweak” is misleading - what kind of mechanical tweak are we considering? Furthermore, extensive research exists on the influence of fatigue on pitching velocity, as well as grip and the influence on spin rate, efficiency and velocity. These are all things that could lead to changes in velocity and are not controlled in this study.

Yes, we agree that grip, fatigue, and lots of other factors affects the pitching velocity. We used too many assertions. After lots of discussions, we decided to remove the “practical applications” subsection and feel the quality of the paper will be better doing so.

Reviewer #2: The aim of study was to predict the outcomes that explain the pitching velocity in adult baseball pitchers. The measurements were age, height, weight, and some field tests including counter movement jump (CMJ), 20 kg-loaded CMJ, 10 m and 30 m sprint time, and maximum internal/external rotation angle of the throwing shoulder. The authors revealed that height, the ratio of the sprint time (10 m / 30 m), and the ratio of CMJ (unloaded / loaded) could predict pitching velocity. Pitching velocity is one of key factors to put out a butter for baseball pitchers and it is important to take the measure of the ability to throw a fastball for coaching staff, scout, and trainer and so on.

However, I have concerns on the followings; (1) a lack of novelty (previous studies have already reported similar results), (2) the use of low costs equipment and the ease of set up are insufficient as a claim of this study, (3) the rationale for the selection of measurements is insufficient, (4) the methodological descriptions of each measurement were not written in detail, and (5) the superficial discussion, especially about the ratio of loaded CMJ/ CMJ.

General comments

1. The authors’ selection on these measurements to predict pitching velocity are not justified: This manuscript does not mention the rationale that these measurements were appropriate to predict pitching velocity among field tests; and most of these measurements were already examined in the previous studies. Furthermore, the value of adjusted R2 was low, which does not fully support the authors’ conclusion. Therefore, unfortunately, it is difficult to find the novelty and relevance of this manuscript.

Yes, we agree most measurements were examined in previous studies, however, most previous studies are done in the lab setting with expensive equipment which not everyone has access to. We try to find the field tests possible to perform with mobile, affordable equipment in the short period of time so can be performed in most tryouts. The tests we performed can be done in a baseball field or any gym with 30 meters length and examine 60 athletes in 2 hours. R2 is mild, but p = 0.0003, so confidence level is very high, and the model is reliable.

In the study “Relationships between ball velocity and throwing mechanics in collegiate baseball pitchers” by Werner et. al, they conclude that “Body mass and 9 temporal and kinematic parameters related to pitching mechanics combine to account for **68%** of the variance in ball velocity for a collegiate population of athletes.” That also says there are some factors affecting pitching velocity yet to be explored for pitching velocity. Thus, our model with inputs from field tests explains **23%** of the variance in pitching velocity, seems reasonable and helps solve part of the puzzle.

2. I suggest that this manuscript should examine whether each CMJ parameters (or speed test parameters) can explain pitching velocity. For example, as mentioned in the methods, CMJ and loaded CMJ were good indicator for the ability of explosive power exertion relate to pitching velocity. If each CMJ parameter relate to pitching velocity, CMJ and loaded CMJ tests were good assessment for pitching velocity. Thus, authors should examine the relationship between CMJ and pitching velocity in detail.

We did. It was explained in the Result section. Loaded CMJ and CMJ were still in the model after stepwise multi-regression. But they were eliminated in the multicollinearity testing process, meaning that they are highly correlated with the loaded CMJ/CMJ ratio and will boost the R2 falsely if included.

3. There are lack of clarities in descriptions of methods. For example, it is not clear whether the shoulder ROM was active or passive, and whether the posture during measurements was sitting or lying. Furthermore, the information about Rapsodo setup and number of pitches during trial or warming up are not described. Please revise the method section to provide much more details about the experiment.

Shoulder ROM was passive, and posture was lying. The setup for Rapsodo and more warmup procedure are added to the Velocity sub-section, thank you for reminding.

Minor comments

1. The authors should space between values and unit.

Revised, thanks for pointing it out.

2. The “throwing velocity” should be changed to “pitching velocity”.

Throwing velocity is changed to pitching velocity, thank you.

3. References should be cited as “xxx et al. (20xx)…”.

References updated, thank you.

4. Title: The title is not supported by results.

There is no one dominant factor in baseball pitching, A lot of factors add up to a pitcher’s velocity. We present that field tests correlate to a pitcher’s velocity at 23%, and the confidence level of the model is very high.

5. Intro: L15 lower extremity → upper extremity or throwing arm?

It is lower extremity we refer to, and it is very important to pitching velocity. There were research on correlation of lower extremity and pitching velocity before and we cited two papers as well.

[7]	J. A. J. Guido and S. L. Werner, “Lower-Extremity Ground Reaction Forces in Collegiate Baseball Pitchers,” *The Journal of Strength & Conditioning Research*, vol. 26, no. 7, pp. 1782–1785, Jul. 2012, doi: 10.1519/JSC.0b013e31824e1211.

[8]	“Relationship Between Vertical Jump Force and Pitching Velocity,” *Driveline Baseball*, Sep. 08, 2016. https://www.drivelinebaseball.com/2016/09/examining-the-relationship-between-vertical-jump-force-and-velocity/ (accessed Jun. 22, 2021).

6. Methods: L49 The participants demographics such as their age, height, and weight were not provided.

Since Taiwan is not very international regarding the athletes living here, participants in this tryout are very close in age, height, and weight. We’ve added that they are all Asian male in the sentence, thanks for reminding.

A total of 64 Asian male pitchers (age: 23.9 ± 2.8 years; height: 180.3 ± 5.9 cm, weight: 81.4 ± 10.9 kg)

7. Results: The results of two ratios about CMJ and sprint time were important factors that explain the pitching velocity. Thus, authors should describe the detailed analysis of CMJ and sprint time (e.g. height, time, and correlation between the values of ratio and pitching velocity).

We did explain it in the discussion section. On the other hand, we do not want to overstate it. We did present the result objectively with some hypothesis.

8. Discussion: L132 cose → cost

Corrected, thank you.

9. References: 17 The journal is Res Q Exerc Sport?

Corrected, thanks for pointing it out.

6. PLOS authors have the option to publish the peer review history of their article (what does this mean?). If published, this will include your full peer review and any attached files.

If you choose “no”, your identity will remain anonymous but your review may still be made public.

Do you want your identity to be public for this peer review? For information about this choice, including consent withdrawal, please see our Privacy Policy.

Reviewer #1: Yes: Mike Sonne

Reviewer #2: No

[NOTE: If reviewer comments were submitted as an attachment file, they will be attached to this email and accessible via the submission site. Please log into your account, locate the manuscript record, and check for the action link "View Attachments". If this link does not appear, there are no attachment files.]

While revising your submission, please upload your figure files to the Preflight Analysis and Conversion Engine (PACE) digital diagnostic tool, https://pacev2.apexcovantage.com/. PACE helps ensure that figures meet PLOS requirements. To use PACE, you must first register as a user. Registration is free. Then, login and navigate to the UPLOAD tab, where you will find detailed instructions on how to use the tool. If you encounter any issues or have any questions when using PACE, please email PLOS at figures@plos.org. Please note that Supporting Information files do not need this step.
